# New-Onset Primary Hypothyroidism in a 14-year-old Girl Presenting as Hemorrhagic Shock From Severe Menorrhagia

**DOI:** 10.1210/jcemcr/luaf098

**Published:** 2025-06-19

**Authors:** Grace Arias, Jaya Parulekar, Annalise Sara Jacobs, Emily Kristen Sims

**Affiliations:** Center for Diabetes and Metabolic Diseases, Indiana University School of Medicine, Indianapolis, IN 46202, USA; Center for Diabetes and Metabolic Diseases, Indiana University School of Medicine, Indianapolis, IN 46202, USA; Center for Diabetes and Metabolic Diseases, Indiana University School of Medicine, Indianapolis, IN 46202, USA; Center for Diabetes and Metabolic Diseases, Indiana University School of Medicine, Indianapolis, IN 46202, USA

**Keywords:** hemorrhagic shock, menorrhagia, hypothyroidism

## Abstract

Hypothyroidism can lead to menstrual irregularities due to its effects on the hypothalamic-pituitary-ovarian axis. Considering this correlation, we present a case of a 14-year-old female in critical condition with hemorrhagic shock from menorrhagia. Initial workup discovered severe anemia with a hemoglobin of 1.8 g/dL (18 g/L) (reference range, 12.0-15.0 g/dL; 120-150 g/L) and profound primary hypothyroidism with a TSH level of 423.7 μU/mL (423.7  mIU/L) (reference range, 0.40-4.2  mIU/mL) and an undetectable free thyroxine of <0.2 ng/dL (<2.5 pmol/L) (reference range, 0.6-1.5 ng/dL; 7.7-19.3 pmol/L). This was confirmed on repeat laboratory testing along with an elevated thyroid peroxidase antibody level of 19.6 IU/mL (reference range, 0.0-9.0 IU/mL) and elevated antithyroglobulin antibody level of 5.0 IU/mL (reference range, 0.0-4.0 IU/mL). She was diagnosed with primary hypothyroidism from autoimmune thyroiditis. Even with blood transfusions, her heavy menstrual bleeding persisted. Thyroid hormone replacement therapy was initiated with oral levothyroxine 50 μg (0.84 μg/kg) once daily, along with high-dose combined oral contraception, with clinical improvement. This severe case presentation illustrates the link between menorrhagia and hypothyroidism in pediatric populations, highlighting the need to address this association in adolescents and ensure optimal care for these patients.

## Introduction

Autoimmune thyroiditis is the most common cause of acquired hypothyroidism in children and adolescents [1]. The incidence of autoimmune thyroiditis during adolescence is 1% to 2% [2]. Hypothyroidism affects various bodily functions with common presenting symptoms of hypothyroidism in children and adolescents including poor growth velocity, decreased energy, declining school performance, constipation, and dry skin [3]. Hypothyroidism can also lead to menstrual irregularities, including menorrhagia or excessive menstrual bleeding, due to its effects on the hypothalamic-pituitary-ovarian axis. As such, the *International Journal of Gynecology and Obstetrics* recommends screening for the presence of an endocrine disorder in patients with menorrhagia [4]. Given the critical presentation of our pediatric patient with menorrhagia due to severe primary hypothyroidism, our case highlights the need to address this association in adolescents and ensure optimal care for these patients.

## Case Presentation

A 14-year-old female with no known medical history was admitted to the pediatric intensive care unit in hemorrhagic shock due to menorrhagia for 3.5 weeks. Menarche was at age 12 years and was chronically characterized by prolonged bleeding. She reported irregular menses occurring every 3 weeks with an average length of 2 weeks of daily bleeding. She required between 3 and 5 heavy-flow pads per day, often experiencing overflow bleeding. She also had a history of fatigue, cold intolerance, and constipation for at least 2 years. Relevant objective measurements on presentation, including vital signs, height, and weight on admission are included in Table 1. On examination, she was pale with pitting edema of her bilateral lower extremities and fine pulmonary crackles on lung examination. She was Tanner stage IV for breast development. No enlarged thyroid gland, thyroid nodules, or cervical lymphadenopathy were appreciated. Notably, family history was significant for hypothyroidism in the patient's mother.

**Table 1.** Height, weight, BMI, and vital signs at presentation

Height (inches)	60
Weight (kilograms)	59.1
Body mass index	25.4
Temperature (Fahrenheit)	86.6 °
Heart rate (beats per minute)	102
Blood pressure (mm Hg)	85/72
Respiratory rate (breaths per minute)	18
Oxygen saturation (%)	88

## Diagnostic Assessment

Initial workup discovered severe anemia with a hemoglobin (Hgb) of 1.8 g/dL (18 g/L) (reference range, 12.0-15.0 g/dL; 120-150 g/L) necessitating resuscitation with blood products. Abdominal and pelvic computed tomography showed a large endovaginal clot and thickened endometrium. Beta-human chorionic gonadotropin was negative. Hematologic evaluation revealed platelets of 195 K/µL (195 ×10^9^/L) (reference range, 150-450 K/µL; 10^9^/L), Prothrombin time of 12.1 seconds (reference range, 10.0-14.1 seconds), International Normalized Ratio of 1.08 (reference range, 0.75-1.26), activated partial thromboplastin time of 23.9 seconds (reference range, 24.6-38.4 seconds), factor VIII 282% (reference range, 60%-138%), and factor IX 109% (reference range, 43%-155%). A comprehensive list of anemia-related laboratory results can be found in Table 2. Further workup with thyroid function tests revealed a TSH of 423.7 μU/mL (423.7 mIU/L) (reference range, 0.40-4.2 mIU/mL) and an undetectable free thyroxine of <0.2 ng/dL (<2.5 pmol/L) (reference range, 0.6-1.5 ng/dL; 7.7-19.3 pmol/L). These results were confirmed on repeat laboratory testing with additional findings of an elevated thyroid peroxidase antibody level of 19.6 IU/mL (reference range, 0.0-9.0 IU/mL) and elevated antithyroglobulin antibody level of 5.0 IU/mL (reference range, 0.0-4.0 IU/mL), consistent with primary autoimmune hypothyroidism. Given the elevated TSH value as a clear cause for her findings, additional endocrine workup laboratory tests such as prolactin, LH, FSH, estradiol, and total testosterone were not obtained, with a plan to pursue further endocrine workup if symptoms did not resolve after levothyroxine treatment. Despite receiving blood product resuscitation, the patient continued to experience heavy vaginal bleeding and persistent severe anemia. The patient was treated with high-dose oral contraception with ethinyl estradiol-norgestimate and daily ferrous sulfate with continued fluctuations in hemoglobin. Thyroid hormone replacement therapy was initiated with a subtherapeutic dose of oral levothyroxine at 50 μg (0.84 μg/kg) once daily with improvement in clinical status. She also required multiple doses of IV conjugated estrogen and tranexamic acid to control her bleeding. Her menorrhagia slowly subsided over multiple days with eventual cessation of vaginal bleeding. The patient and family were instructed to continue this dose of levothyroxine for 2 weeks with a plan to escalate levothyroxine to therapeutic dosing over time.

**Table 2.** Anemia-related labs collected upon presentation

Laboratory test	Value	Conventional units (SI units)	Reference ranges
White blood cell count	30.4	K/µL	3.6-10.6
Hemoglobin	1.8	g/dL (g/L)	12.0-15.0 g/dL (120-150 g/L)
Hematocrit	5.4	%	35-49
Mean corpuscular volume	90	fL	81-99
Mean corpuscular hemoglobin concentration	34.4	g/dL	32-36
Red cell distribution width	14	%	11.5-14.5
Platelet count	195	K/µL (10^9^/L)	150-450 K/µL or 10^9^/L
Prothrombin time	12.1	seconds	10.0-14.1
International Normalized Ratio	1.08		0.775-1.26
Activated partial thromboplastin time	23.9	seconds	24.6-38.4
Factor VIII assay	282	%	60-138
Factor IX assay	109	%	43-155
von Willebrand activity screen	83	%	54-137
Platelet function analyzer in collagen/ADP	76	seconds	54-117

## Treatment

Upon initial presentation, this patient received 4 units of packed red blood cells and 1 unit of fresh frozen plasma. Given that the source of her anemia was found to be uterine in origin, she was also given ethyl estradiol-norgestimate 35 μg-0.25 mg oral tablets 3 times daily for 4 doses along with a daily ferrous sulfate. After attempts to reduce the frequency of her oral contraception, her vaginal bleeding worsened. This prompted the administration of IV conjugated estrogen 25 mg every 4 hours for 3 doses along with tranexamic acid 650 mg 3 times daily for 5 doses. Thyroid replacement therapy was concomitantly initiated with a subtherapeutic dose of 50 μg (0.84 μg/kg body weight) daily. Although we typically start children with profound hypothyroidism on 25-μg dosing of levothyroxine to avoid iatrogenic symptoms of hyperthyroidism, a more aggressive 50-μg dose was deemed reasonable in the severe clinical setting of refractory menstrual bleeding resulting in hemorrhagic shock. By time of discharge, the patient was clinically stable on levothyroxine 50 μg daily and ethinyl estradiol-norgestrel 30 μg-0.3 mg orally every eight hours with a plan to empirically escalate levothyroxine dosing to 100 μg after several weeks if the patient was tolerating the medication without issue.

## Outcome and Follow-up

**Figure 1.**
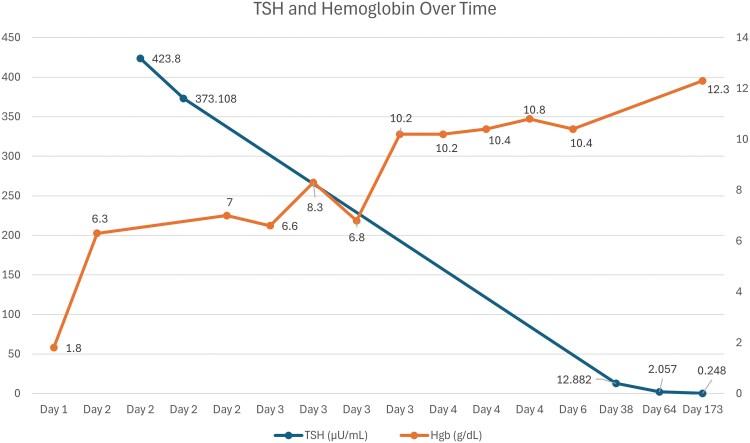
THS and hemoglobin levels plotted over time. Serial measurements of TSH (blue line) and hemoglobin (Hgb, orange line) levels in our single patient starting from day of hospitalization admission (day 1) and continuing through subsequent days. TSH levels showed a decreasing trend, while Hgb levels gradually increased over time following initiation of levothyroxine therapy. TSH is plotted on the left y-axis (mcU/mL) and Hgb on the right y-axis (g/dL). The x-axis represents time in days.

After the multimodal treatment regimen described above, Hgb by time of discharge had risen to 10.4 g/dL (104 g/L). Upon posthospitalization follow-up 1 month later, the patient reported improved energy while taking 100 μg (1.69 μg/kg) of levothyroxine daily. She continued to have menstrual suppression with once-daily adherence to ethinyl estradiol-norgestrel 30 μg-0.3 mg oral tablet for continuous cycling with plans to transition to monthly cycles as her hemoglobin normalized. Approximately 6 weeks after initial presentation, her TSH level had improved but remained elevated at 12.8 μg/mL with free thyroxine normalized to 0.9 ng/dL. Her Hgb also normalized to 12.3 g/dL (123 g/L). An additional increase in levothyroxine dosage to 112 μg (1.89 μg/kg) was made at this appointment with subsequent normalization of thyroid function. A graphical representation of the patient's change in TSH and Hgb over time can be found in Fig. 1.

## Discussion

Menorrhagia and anemia can both be signs of hypothyroidism [3]. In fact, hypothyroidism is associated with various menstrual irregularities, including menorrhagia, due to its effects on hormonal balances. The interaction between TSH, FSH, and LH receptors provides a mechanistic link between hypothyroidism and menorrhagia. All 3 hormones consist of a common α subunit and specific β subunits [5]. Elevated TSH found in hypothyroidism can exhibit cross-reactivity with FSH and LH receptors due to structural similarities among these glycoprotein hormones. This cross-reactivity can lead to altered ovarian function and menstrual irregularities. In hypothyroidism, the elevated TSH levels can mimic FSH and LH activity, potentially leading to increased ovarian stimulation and subsequent menorrhagia [6]. Case reports and studies have documented the relationship between hypothyroidism and menorrhagia, particularly in adult literature. In pediatric populations, severe hypothyroidism has also been reported to present with menorrhagia as a primary symptom. Another case report described a 13-year-old girl with severe hypothyroidism and menorrhagia, which resolved with levothyroxine therapy [7]. In our present case, the significant menstrual bleeding experienced by our adolescent girl prompted a thorough investigation, leading to the diagnosis of autoimmune thyroiditis and subsequent initiation of thyroid replacement therapy. Before hypothyroidism was diagnosed, the differential for this presentation was broad, including hematologic and uterine abnormalities. This patient's severe presentation serves as a reminder that a comprehensive clinical and family history is pertinent to establishing an accurate diagnosis of hypothyroidism. Some reports have advocated for low-dose levothyroxine replacement in pediatric populations with profound hypothyroidism [8], with a goal of preventing adverse cardiac events due to normalizing thyroid function too quickly. Here, pediatric endocrinologists often initially replace thyroid hormone with an intentionally low, subtherapeutic dose of levothyroxine that is increased slowly over several months to avoid iatrogenic symptoms of hyperthyroidism such as inattention and hyperactivity, as well as the theoretic risk of more severe pseudotumor cerebri or cardiac failure [9]. Given that the risks vs benefits of this approach have not been robustly tested, we chose a more moderate approach to levothyroxine replacement in this setting, which worked to achieve the goal of symptom control in the context of our patient's refractory anemia and critical condition without subsequent adverse cardiac events.

## Learning Points

- Severe menorrhagia and life-threatening anemia can be a presenting sign of profound primary hypothyroidism.
- Thyroid function should be evaluated in pediatric patients presenting with severe menorrhagia and hemorrhagic shock.
- The expanding evidence linking menorrhagia with hypothyroidism in pediatric patients can inform practitioners to readily screen these patients for thyroid dysfunction, facilitating early detection and management of hypothyroidism in individuals presenting with menorrhagia.
- Starting levothyroxine replacement at a moderate dose in adolescent patients with refractory symptoms aims to achieve symptom control for conditions like resistant menorrhagia.

## Contributors

All authors made individual contributions to authorship. G.A., J.P., A.S.J., and E.K.S. were involved in manuscript development and submission. J.P., A.S.J., and E.K.S. were involved in the diagnosis and management of this patient. All authors reviewed and approved this final draft.

## Data Availability

Data sharing is not applicable to this article as no datasets were generated or analyzed during the current study.
